# Aspects of Inflammation and Oxidative Stress in Pediatric Obesity and Type 1 Diabetes: An Overview of Ten Years of Studies

**DOI:** 10.1155/2012/683680

**Published:** 2012-10-11

**Authors:** Brian Tran, Stacy Oliver, Jaime Rosa, Pietro Galassetti

**Affiliations:** ^1^Insitute for Clinical and Translational Science, University of California, 843 Health Science Road, Irvine, CA 92697, USA; ^2^Department of Pediatrics, School of Medicine, University of California, 100 Theory, Irvine, CA 92697, USA; ^3^Department of Pharmacology, School of Medicine, University of California, 360 Med. Suzge II, Irvine, CA 92697, USA

## Abstract

Obesity and type 1 diabetes (T1DM) are the two most common conditions of altered metabolism in children and adolescents. In both, similar long-term cardiovascular complications are known to occur, mediated in large part by underlying inflammatory and oxidative processes whose biochemical details remain relatively unclear. Through a series of experiments in these patient populations, over the last decade our laboratory has clarified a number of key issues in this field. Interestingly, while obese and type 1 diabetic children often differed in the specific type and magnitude of molecular alterations, in both groups a clear exaggeration of inflammatory and oxidative activation was detected when compared to healthy, age-matched controls. Our main findings include definition of resting and exercise-induced cytokine patterns and leukocyte profiles, patterns of activation of immune cells *in vitro*, and correlation of the magnitude of observed alterations with severity of obesity and quality of glycemic control. Further, we have identified a series of alterations in growth factor profiles during exercise that parallel inflammatory changes in obese children. This paper offers a concise overview of the salient results from this decade-long research effort.

## 1. Introduction

In a time when remarkable scientific advances have occurred in all fields of medicine, it seems sometimes anachronistic that in some areas, in which awareness of a given pathological condition and general knowledge of its pathophysiology have been established for decades, improvements in prevention, management, and avoidance of complications have not been as rapid and complete as could have been expected. This is especially true for conditions of altered metabolism in pediatric populations, such as obesity and diabetes, in which lack of a deep and thorough understanding of multiple disease aspects still prevents the widespread, effective control of health care and social problems associated with these conditions. In children and adolescents, obesity and type 1 diabetes (T1DM) are by far the two most common dysmetabolic conditions. While in recent decades obesity has been increasing at alarming rates in industrialized countries across all age groups, this phenomenon has been especially pronounced in children and adolescents. The obvious threat to which these populations are exposed is that if the condition is not corrected later in life, which unfortunately in the large majority of cases is not the case, onset and progression of related cardiovascular complications will occur at a proportionally earlier age, with enormous estimated social, health care, and emotional costs. Among obesity complications is of course also type 2 diabetes (T2DM). While this condition accounts for ~90% of all diabetes cases and is also occurring at much greater rates in pediatric ages than was used to only 20 years ago, it is still much less common than T1DM among children and adolescents. Paradoxically, while the recent increase in T2DM has been explained with the parallel obesity epidemic, the prevalence of T1DM has also been increasing in recent years, for reasons that remain unclear. Obesity and T1DM share a number of common features. Most importantly, in both conditions, long-term complications include severe impairment of the cardiovascular system. While this association has been clearly established empirically, much still remains to be learned about the specific pathogenetic mechanisms regulating the development of these complications. Consensus exists among the leading research groups in this field that a major role is played by chronic subclinical levels of inflammation and oxidative stress which, in both conditions, through slow but persistent damaging action on the endothelial surface, eventually result in stiffening, narrowing, and occlusion of arteries of various caliber in a broad range of tissues. Inflammation and oxidative stress, however, are very extensive and complex processes encompassing the coordinated action of hundreds and possibly thousands of cellular and molecular mediators, including dozens of subtypes of immune cells (with shifting patterns of surface markers of activation), hundreds of cytokines, chemokines, growth factors, and their related receptors and binding proteins. Which components of this intricate network are activated at any given time, for how long, and to what degree, at any stage of obesity and T1DM, remains very poorly defined. Further, it is unclear to what degree any of the reported levels of exaggerated inflammation or oxidative stress are due to the presence of obesity or T1DM per se, or to an acute exacerbation of a specific aspect of each condition, such as acute hyperglycemia in T1DM or hyperlipidemia in obesity. To add a further layer of complication, most of what we know about interaction of metabolic dysregulation and inflammation/oxidative stress in obesity and T1DM is derived from studies in adults and may therefore not be applicable to pediatric populations in which the endogenous metabolic milieu changes rapidly and repeatedly during growth and development. Our group has therefore performed, over the last decade, a number of studies focused on the attempt to clarify key aspects of this interaction in pediatric populations. This paper is aimed at providing a broad overview of the main finding of these prolonged efforts.

 Before we even start reporting our findings in these specific populations, however, it should be clear that this is only part of a broader context, in which inflammation, oxidative stress, and their interaction with exercise modulate health status in many more subject populations with related dysmetabolic conditions. Pediatric prediabetic states, for instance, may present with significant inflammatory alterations; in pre-type 2 diabetes, the enormous increase in circulating insulin may act by itself as a major inflammatory modulator, independent of obesity status [1]. In pre-type 1 diabetes, when autoantiobodies start increasing, a massive surge in inflammatory processes is preparing to explode which, if correctly identified in its exact time frame, holds great promise for preventive interventions that may block the evolution into frank diabetes [2]. Finally, even within the normal BMI range, positions close to the overweight range may be associated with less dramatic, but still clinically relevant differences in inflamamtiory status [3]. While all these conditions are important and definitely need to be explored in detail, their comprehensive evaluation is beyond the scope of this paper, which we will therefore keep focused on the two quantitatively largest pediatric dysmetabolic conditions, T1DM and obesity.

## 2. Inflammation in T1DM

Type 1 diabetes mellitus (T1DM) has long been associated with the development of cardiovascular disease (CVD). Numerous epidemiological studies have observed that diabetic subjects had extremely high risks of developing atherosclerosis, acute coronary events, and stroke relative to the general population [4, 5]. This risk is attenuated with very good glycemic control (DCCT); however, even very well-controlled patients retain considerably higher cardiovascular risk as compared to healthy matched controls.

Multiple lines of recent evidence indicate that the biochemical link between diabetes and later development of CVD includes the two parallel and related processes of increased inflammation and oxidative stress. T1DM is characterized as an inflammatory disease from several points of view. First, an acute, intense inflammatory reaction causes the very onset of the disease via lymphocyte-mediated destruction of pancreatic beta cells. After this first major inflammatory episode is resolved, a chronic state of whole-body low-grade inflammation appears to persist, which is periodically exacerbated by hyperglycemic fluctuations. In fact, elevated markers of inflammation [6], immune activation [7], and oxidative stress [8, 9] have been observed in the T1DM population. All of these factors are heavily associated with the initiation and progression of atherosclerosis and CVD [10]. However, the exact pathogenesis of these alterations is not well understood. This issue becomes particularly relevant when considering the disease in children, where the alterations have potential implications in the complex interaction of early onset of disease symptoms, growth, and development. Work in our laboratory over the last several years has continued with this line of inquiry; we have conducted a number of experiments designed to help isolate and characterize factors involved in the T1DM child's inflammatory state.

In an early study with 12 T1DM children and healthy controls, we observed a distinctly altered inflammatory cytokine profile for T1DM children. In particular, the T1DM group displayed markedly elevated interleukin 6 (IL-6), a molecule considered a classic proinflammatory mediator, but which, in specific context, has to be also hypothesized to exert opposite, antiinflammatory effects [11]. This finding was later confirmed in a larger study on 49 T1DM and 42 healthy controls, in which a more comprehensive inflammation and oxidative stress biomarker panel was performed. The T1DM group displayed not only elevated levels of IL-6, but also significantly higher plasma myeloperoxidase (MPO), an oxidative stress marker derived from neutrophils, monocytes, and macrophages. The latter observation is in agreement with prior studies indicating that chronic excessive activation of these cells lines is involved in the progression of atherosclerosis and cardiovascular disease in a number of dysmetabolic states [12, 13]. Additional evidence that inflammatory processes may be exaggerated in T1DM can also be found in a recent study that focused on observing gene expression of proinflammatory cytokines and chemokines in leukocytes following T-cell receptor and Fc receptor stimulation [14]. Leukocytes from T1DM children displayed exaggerated gene expression for TNFSF 5, 7, and 9, CCL8, and CXCL10 compared to healthy controls.

One of the main issues driving our experimental design was the relative contribution of presence of diabetes per se, versus periodic hyperglycemic exacerbations, to the overall inflammatory status in these patients. Hyperglycemia is known to induce an acute state of inflammation, both in healthy and in diabetic subjects [15]. It had not been demonstrated, however, if this increased inflammatory state would persist after the hyperglycemic episode was resolved. This is especially relevant to T1DM children with poor glycemic control, where repeated hyperglycemic peaks are particularly common. In these subjects, the inability to fully correct for the proinflammatory effect of hyperglycemic episodes may result in chronically elevated inflammation, thus worsening its long-term damaging effects on the vascular system. We therefore measured a number of immune-modulatory cytokines in children with T1DM who were either normo- or hyperglycemic. Among these, IL-1*α*, IL-4, and IL-6 were significantly elevated in the hyperglycemic group not only while their glucose was high, but also for at least 2 hours after hyperglycemia had been corrected [16]. The presence of this sustained inflammatory state stressed the importance of preventing as opposed to correcting hyperglycemia and implied that additional therapeutic approaches, such as anti-inflammatory regimens, targeting this state in poorly controlled T1DM may be necessary to help prevent vascular complications.

**Figure 1:**
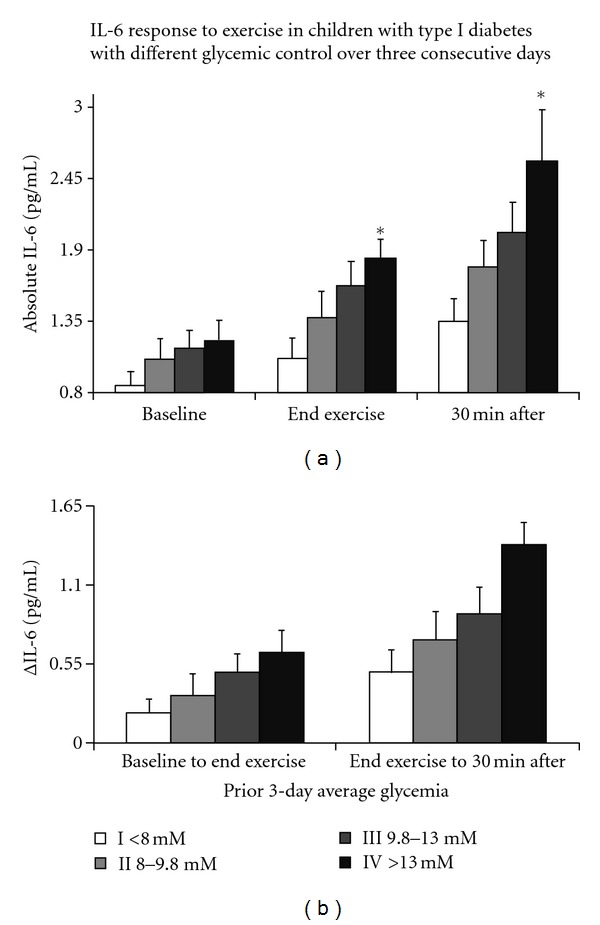
Plasma interleukin-6 (IL-6) in 47 children with type 1 diabetes before, at the end of, and 30 min after a standardized 30 minute intermittent exercise challenge (a) and exercise-induced IL-6 increments (b). While all children exercised in euglycemic conditions (and had been euglycemic for at least the two hours preceding exercise), they differed in average glycemic control during the previous three days and were therefore subdivided in four groups (11-12 subjects each) with increasing mean prior 3-day glycemia. Children in the group with the lowest mean glycemia (<8 mM, only slightly greater than comparable healthy controls) displayed the lowest IL-6 values at all time points; with greater mean prior glycemia, IL-6 values became progressively higher. Data are group means ± SE. *P < 0.05 <8.0 mM group I.

A logical extension of the above experiments became the characterization of the nature of the hyperglycemic states (severity, durations, or distance in the past) that led to subsequent sustained inflammation. In a previous study, we divided 29 T1DM children in to 4 subgroups, based on their spontaneous morning glycemic level; we then normalized blood glucose in all subjects, kept them euglycemic for at least 2 hours, and then measured plasma IL-6. Not surprisingly, we observed that the group with the highest prior morning plasma glucose (>300 mg/dL) also had the highest IL-6 concentrations; the other three groups (prior glycemia of 200–300, 150–200, and <150 mg/dL, resp.) displayed progressively lower IL-6 levels, the lowest being identical to healthy controls. A subsequent study expanded these findings by incorporating the effect of prior hyperglycemia not only in the morning of the measurements, but also for the full previous three days. Participants wore a continuous glucose monitoring system recording glycemia every 5 minutes for a 3-day period while continuing their normal insulin regimen and conducting normal life activities (Figure 1). This allowed us to obtain additional information, including depth, duration, and repetition pattern of each hyperglycemic episode, as well as total time spent above arbitrary hyperglycrmic thresholds (i.e., 11 mM, the clinical definition of postprandial hyperglycemia). Among these variables, the average glycemia over the whole 3-day period correlated best with IL-6 levels at the end of the continuous measurements, after all subjects' glycemia had again been normalized for several hours. Again, children with the highest mean prior 3-day glycemia had the highest IL-6 levels and progressively lower IL-6 as their prior glycemia approached the physiological profile. Within subjects with similar mean 3-day glycemia, a greater proinflammatory effect was observed in those with the highest hyperglycemic peaks, indicating that optimal management strategies in these patients must be aimed not only at limiting the overall occurrence of hyperglycemia, but also at preventing its most dangerous characteristics (for example, the same overall amount of postprandial hyperglycemia can be achieved without a very high hyperglycemic peak if insulin administration is timed carefully and carbohydrate ingestion is spaced over a longer time).

One well-known therapeutic approach to reduce whole-body inflammation is exercise training. In the context of T1DM, the benefits are obvious: as stated previously, the potential risk for the development of CVD via a sustained state of inflammation is attenuated. Interestingly, a reduction in systemic inflammatory status occurs with long-term training [17, 18], while, somewhat paradoxically, individual bouts of exercise are in contrast acutely proinflammatory. This would normally not be an issue in the healthy subject, where the exercise-induced inflammation is well controlled, but in T1DM children, in whom our experiments have shown to be unable to fully correct for hyperglycemia-induced inflammation, the presence of this proinflammatory effect may mitigate the benefits of exercise or may even lead to deleterious effects over time. To address this issue, we conducted a number of experiments to clarify the effect of exercise on T1DM children.

Central to our exercise studies is the exercise protocol, which was specifically developed in our laboratory to both normalize work rate and maximize the physiological adaptive response, while also simulating real-life spontaneous physical activity. The test consisted of performing, on a stationary bike, a series of ten 2-minute exercise bouts at 80% of individual maximal aerobic capacity (VO2max, determined during a preliminary separate test), separated by 1-minute intervals. This sequence made it easier for children to tolerate the 30-minute challenge and produced a similar set of response as could be elicited by the stop-and-go pattern of a soccer or basketball game. This exercise protocol, now having been applied thousands of times to a variety of different experimental settings and in children with numerous health conditions, has been instrumental in ensuring that the physical exertion from exercise is normalized across different subjects.

Inflammation is a multifaceted condition, with many aspects mediated via activation of immune cells. Indeed, leukocyte counts physiologically increase acutely by ~50% during exercise. This effect seems not to be altered by the presence of diabetes, as demonstrated by a recent study from our laboratory in which we measured circulating leukocytes (neutrophils, lymphocytes, and monocytes), before and after the exercise challenge described above, in 45 healthy and 16 T1DM children. In both groups, all leukocyte measurements, including total and the subtypes, were significantly elevated at end exercise and returned to near baseline at 30 minutes after exercise. In pediatric diabetes therefore, an increased contribution of circulating immune cells to systemic inflammation seems to be mediated, rather than by an increase in their number, by their levels of proinflammatory activation.

While several studies have measured cytokine response to exercise, especially IL-6, considerable inconsistency exists in the published literature as to the extent, and in some cases presence at all, of these responses (e.g., an increase or decrease in a specific cytokine from exercise). Coincidentally, many studies that looked into this effect simply measured two points (usually at baseline and after exercise), with conclusions that were based off of a linear interpolation of those points. Should cytokine levels actually fluctuate in a nonlinear manner during physical activity, two time points could have provided an incomplete view of the overall response to exercise. Thus, our group conducted a study to better clarify this issue [19]. Twenty one T1DM children and age-matched healthy controls underwent our standard exercise challenge. In this experiment, however, additional blood samples were drawn every 6 minutes during exercise and 4 times in 15-minute intervals after exercise. Testing revealed a relatively conserved, non linear pattern across various cytokines and chemokines. The control subjects generally exhibited an initial drop in circulating inflammatory mediators during exercise (possibly due to binding of existing molecules to the acutely increase numbers of circulating leukocytes), followed by a measurable rise at end of or after exercise (possibly due to new cytokine/chemokine molecules being secreted in response to exercise stimulus). T1DM subjects, for the majority of cytokines and chemokines, did not display the initial drop during exercise and instead exhibited an exaggerated and accelerated profile of inflammatory activation, with larger increases of mediators that peaked more rapidly during exercise. This was most clearly seen in the cytokines IL-6, tumor necrosis factor-*α* (TNF-*α*), and the chemokines monocyte chemoattractant protein-1 (MCP-1) and macrophage inflammatory protein-1*α* (MIP-1*α*). The practical meaning of this finding is that for the whole duration of exercise, at least in this particular format, T1DM children were exposed to a clearly more proinflammtory milieu, with greater concentrations of both proinflammatory mediators and of chemoattractants/activators for monocytes, known to play a key role in the early development of vascular atherosclerotic lesions.

## 3. Inflammation and Pediatric Obesity

Along with T1DM, obesity is among the most common dysmetabolic disorders in children. Modern society's increasingly sedentary lifestyles and growing ease of overnutrition have contributed to the prevalence and incidence of excessive adiposity, which has consistently risen in the US throughout the past four decades. In the latest NHANES conducted by the CDC, about 17% of children aged 9–17 were observed to be clinically obese [20], and other reports indicate that the prevalence may be even higher in the US population overall and certainly is within certain populations and at risk minorities [21]. Early-onset obesity has been clearly linked to a very high likelihood of life-long permanence of obesity, resulting in an exponential increase in the risk for CVD and other complications. The biochemical details and in-depth metabolic characteristics of pediatric obesity, however, which may differ substantially from adults and are at the very base of our ability to develop effective preventive and therapeutic strategies, remain very poorly understood. In this context, in our laboratory, we have therefore focused on identifying a number of physiological and pathological aspects of pediatric obesity, particularly as related to exercise.

The previously mentioned leukocytosis response study also included overweight children (BMI% > 85) [22]. These subjects exhibited a pattern and magnitude of exercise-induced leukocytosis that was similar to healthy and T1DM children (i.e., all leukocyte subpopulations increased similarly in response to exercise and returned to near-baseline levels within 60 min after exercise cessation); as the study also included yet another group of children (with asthma), who also displayed a similar unaltered response pattern, the data indicate that leukocyte responses to exercise are a highly conserved adaptation mechanism that remains unaltered across a wide range of conditions. It should be noted, however, that the similarity in baseline leukocyte counts between overweight and healthy controls, observed in this study, was somewhat in contrast with a previous study, in which overweight children had higher baseline counts [23]. We believe that while this discrepancy may be due to differences in the severity of overweight/obesity, more severely obese children, therefore, may start exercising with higher basal leukocytes, and even maintaining a normal leukocyte response to exercise, they may be exposed to higher leukocyte levels at all times during and immediately after exercise, likely resulting in parallel increases in other indices of inflammation.

Additional support for this last concept was in fact provided by other studies in which multiple inflammatory mediators were measured in obese children during exercise. For instance, significantly larger levels of baseline and exercise-induced IL-6 were observed in overweight children by McMurray et al. [24]. A later study using our standardized exercise challenge, in which a broad panel of cytokines were measured at multiple time points during exercise, further supported this notion with significant elevations of TNF-*α* and IL-2 and parallel, marked elevation (albeit not statistically significant) of IL-6, IL-4, IL-5, IL-8, IL-10, andIL-13in obese subjects (BMI% > 95) [25]. In addition to inflammatory cytokines, key oxidative stress markers (MPO and F_2_-isoprostanes) were also observed to be elevated in obese children both at baseline and throughout exercise [14, 26]. These studies suggest that chronic inflammation and oxidative stress, which in adults have been suggested as the biochemical link between obesity and its cardiovascular complications, are already markedly activated when obesity is established at a very early age.

In obesity, many changes in the inflammatory and oxidative response to exercise occur in the broader context of a more complex adaptive response which also include a series of hormones regulating availability of energy substrates and systemic anabolism (insulin, glucocorticoids, catecholamines, glucagon, and growth factors). While in adults these processes, if altered, are therefore likely to only affect exercise performance, in the growing child hormonal alterations, especially within the growth hormone (GH) insulin-like growth factor (IGF-1) axis, may have additional effects on growth and development. Indeed, several prior observations from other laboratories, derived from studies on adult populations, supported the concept that either the presence of obesity or the ingestion of a high-fat meal could reduce the GH response to exercise. In 1970, in fact,Hansen and Johansen [27], and in 1999, Kanaley et al. [28] showed that both subjects with upper-body or lower-body obesity had a significantly lower GH peak during a standardized exercise intervention, as compared to control. In a separate study, Cappon et al. displayed how in young healthy subject who performed an intense, 10 min exercise bout 45 min after ingesting a lipid-rich drink, the GH response was attenuated by over 50%, as compared to an identical exercise test, in the same subjects, performed either in fasting condition or after ingestion of an isocaloric carbohydrate-rich drink [29]. These observations. However, had not been confirmed in children until in our laboratory, using our standard exercise protocol, 25 obese children and healthy controls were studied. We observed that, much like in adults, the obese group displayed significantly lower circulating GH, epinephrine, norepinephrine, and dopamine. Other components of the GH-IGF-1 (IGF-1, IGF-, and GH-binding proteins) axis were not significantly different [30].

To determine whether the alterations in the GH response to exercise might become more pronounced in children with a greater severity of obesity, we repeated the same study on a larger subject pool (48 obese, 42 age-matched controls), with the obese subdivided in three subgroups with increasing BMI percentile (95–97th, 97–98.5th, and >98.5th BMI%) [31]. Interestingly, the more obese the children, the more blunted the GH response to exercise; this effect was also maintained both in early- and late-pubertal subjects.

Independent of obesity status, we also addressed the issue of whether, as shown in adults, a high-fat meal could attenuate exercise-induced GH secretion in children. This point may be very relevant to everyday life, as diet-induced obesity with frequent ingestion of high-fat nutrients is now particularly commonplace with the increased availability and convenience of fast food. A realistic scenario that may result from this trend is the practice of eating high-fat meals before physical activity (e.g., lunch before sports practice). We therefore studied a group of healthy children performing standardized exercise following a high-fat meal or placebo [30]. While basal growth hormone was similar across all subjects, the exercise-induced peak was found to be significantly lower after the high-fat meal. We then repeated the same study in obese children and observed that the combined effect of lipid ingestion and obesity almost completely suppressed the GH response to exercise, that is, reduced it to a much greater extent than could be expected by adding the separate effect of obesity and of fat ingestion alone, suggesting the presence of a synergistic effect of the two conditions [32].

## 4. Conclusions

While the picture of inflammatory and oxidative status in children with obesity and T1DM is by no means complete, we believe that a number of general conclusions can be drawn by our ten-year experience of studies in this field. First and foremost, it is clear that no matter what approach was taken (i.e., which mediators were measured, or what experimental protocol was utilized), differences across groups always reveled exaggerated inflammatory/oxidative activation in the obese and diabetic children as compared to healthy age-matched controls (Table 1).

Key inflammatory cytokines (IL-6, TNF-*α*) were among the biomarkers more consistently elevated; further, immune cells displayed greater reactivity in diabetic subjects, secreting larger amounts of inflammatory mediators in response to standard stimuli. When challenged with exercise, a stimulus that has the peculiar characteristic to exert a long-term anti-inflammatory effect, despite acute, proinflammatory responses to each individual exercise bout, obese, and diabetic children again displayed elevated and accelerated patterns of inflammatory activation. Far from indicating that exercise should be avoided in these children, these data rather indicate the necessity of better understanding the characteristics of each exercise format to be utilized in specific subgroup of individuals. Another clear finding of our studies is that while in both obese and diabetic children inflammatory and oxidative processes appeared consistently elevated, the individual factors that were altered differed across the two patient populations, suggesting complex underlying activating mechanisms. Further, the magnitude of inflammatory activation appeared to be proportional, in obese children, to the severity of obesity, and in T1DM children with the quality of glycemic control. Finally, we observed how inflammatory changes were paralleled by comparable alterations in related signaling systems, especially growth factors, with possible implications in the regulation of overall growth and development. As a whole, our data indicate how a better and more complete understanding of all aspects of the interaction between these two widespread pediatric dysmetabolic conditions, and the components of the underlying inflammatory and oxidative dysregulation, is at the very base of our future ability to successfully prevent, manage, and treat the related devastating cardiovascular complications.

## Figures and Tables

**Table 1:** Synopsis of key inflammatory/oxidative alteration in children with type 1 diabetes and obesity.

Inflammatory/oxidative alteration	Evidence
Pediatric type 1 diabetes mellitus	

Altered inflammatory status	Elevated baseline IL-6 (and other proinflammatory mediators); exaggerated gene expression of TNFSF 5, 7, and 9, CCL8, and CXCL10 following *ex vivo* leukocytic TCR and Fc receptor stimulation; exercise-induced changes in IL-6, TNF-*α*, MCP-1, and MIP-1*α* peak more rapidly and in greater magnitude
Altered oxidative stress	Elevated MPO and F_2_-isoprostanes
Inflammatory status may be induced/exacerbated by hyperglycemic episodes	Elevated IL-1*α*, IL-4, and IL-6 in hyperglycemic T1DM children
Inflammation from hyperglycemia is not fully corrected for following reversion to euglycemia	Progressively elevated inflammatory markers based on past 3-day average glycemia
Inflammation may be mediated by level of immunologic proinflammatory activation, rather than cell numbers	Leukocytosis response is conserved as compared to healthy controls

Pediatric obesity	

Elevated inflammatory status	Elevated leukocytes and baseline and exercise-induced IL-6, TNF-*α*, and IL-2
Altered oxidative status	Elevated MPO and F_2_-isoprostanes
Reduced exercise-induced growth hormone secretion in	
Healthy children	Cappon et al. 1993 [29]
Obese children	Oliver et al. 2010 [26, 31]
Markedly reduced exercise-induced GH secretion in Pediatric obesity following a high-fat meal	Oliver et al. 2012 [32]
